# Dietary approaches to stop hypertension (DASH)-style diet in association with gastroesophageal reflux disease in adolescents

**DOI:** 10.1186/s12889-023-15225-6

**Published:** 2023-02-17

**Authors:** Sara Beigrezaei, Bahareh Sasanfar, Zahra Nafei, Nasrin Behniafard, Majid Aflatoonian, Amin Salehi-Abargouei

**Affiliations:** 1 Nutrition and Food Security Research Center, Shahid Sadoughi University of Medical Sciences, Yazd, Iran; 2 Department of Nutrition, School of Public Health, Shahid Sadoughi University of Medical Sciences, Yazd, Iran; 3 Children Growth Disorder Research Center, Shahid Sadoughi University of Medical Sciences, Yazd, Iran; 4 Mother and Newborn Research Center, Shahid Sadoughi University of Medical Sciences, Yazd, Iran; 5 Yazd Cardiovascular Research Center, Non-communicable Diseases Research Institute, Shahid Sadoughi University of Medical Sciences, Yazd, Iran

**Keywords:** Diet, Dietary approaches to stop hypertension, Gastroesophageal reflux disease, Adolescents, Cross- sectional

## Abstract

**Background:**

Dietary patterns and food items have been associated with gastroesophageal reflux disease (GERD) risk and they have led to conflicting findings. The aim of this study was to determine the association between a dietary approach to stop hypertension (DASH)-style diet with the risk of GERD and its symptoms in adolescents.

**Study design:**

Cross-sectional.

**Methods:**

This study was performed on 5,141 adolescents aged between 13 and 14 years. Dietary intake was evaluated using a food frequency method. The diagnosis of GERD was done by using a six-item GERD questionnaire that asked about GERD symptoms. A binary logistic regression was used to assess the association between the DASH-style diet score and GERD and its symptoms in crude and multivariable-adjusted models.

**Results:**

Our findings revealed that after adjustment for all confounding variables, the adolescents with the highest adherence to the DASH-style diet had a lower chance of developing GERD [odds ratio (OR) = 0.50; 95%CI 0.33–0.75, P_trend_< 0.001)], reflux (OR = 0.42; 95%CI 0.25–0.71, P_trend_=0.001), nausea (OR = 0.59; 95% CI:0.32–1.08, P_trend_=0.05) and stomach pain (OR = 0.69; 95%CI 0.49–0.98, P _trend_=0.03) compared to those with the lowest adherence. Similar results were found for odds of GERD among boys, and the total population (OR = 0.37; 95%CI: 0.18–0.73, P_trend_=0.002, OR = 0.51; 95%CI: 0.34–0.77, P _trend_<0.0, respectively).

**Conclusion:**

The current study revealed that adherence to a DASH-style diet might protect against GERD and its symptoms including, reflux, nausea, and stomach pain in adolescents. Further prospective research is needed to confirm these findings.

**Supplementary Information:**

The online version contains supplementary material available at 10.1186/s12889-023-15225-6.

## Background

Gastroesophageal reflux disease (GERD) is a common chronic and highly prevalent disorder [1] that the prevalence of the disease ranges from 2.5 to 7.8% in east Asia up to 18.1–27.8% in North America [2]. It has been shown that individuals with GERD in childhood and adolescence have a higher risk of developing reflux disorder in adulthood [3]. It seems that multiple environmental and genetic factors might have a role in GERD development [4–6]. GERD has a remarkable impact on quality of life [1] and it is related to a higher risk of Barrett’s esophagus that may progress to esophageal adenocarcinoma, as well [7].

It is essential to recognize the life-style related risk factors, particularly diet to manage GERD at younger ages [8]. Several epidemiologic studies have tried to examine the relationship between GERD symptoms and diet components [9, 10]. Previous studies have found no association between GERD and the intake of fruits, vegetables, fish, red meat, pasta, rice, milk, and potato [11–13]; however, it is reported that some food components like salt and alcohol might adversely affect GERD [14, 15].

Recently, there is growing attention to using dietary pattern approaches in epidemiological studies to evaluate the association between diet and chronic diseases [16]. Dietary patterns account for the complexity of diet and the interaction between foods and nutrients, which can facilitate translation into dietary practice [16, 17].

Dietary approaches to stop hypertension (DASH) diet is one of the well-known dietary patterns that has been associated with the prevention and management of several chronic diseases such as hypertension [18], type 2 diabetes [19], and coronary artery disease [20]. This diet is high in vegetables and fruits, legumes, nuts, and fish, moderate in low-fat dairy products, and low in animal protein, salt, and sweetened beverages [21]. Adherence to the DASH diet can provide a healthy dietary pattern and promote sources of vitamins, minerals, and also antioxidants [22] which in turn might potentially influence gastrointestinal (GI) disorders including GERD [23]. Some previous studies have demonstrated the relationship between the DASH diet and GERD [24, 25]. A study by Gong et al. [24] found that high salt intake, inadequate fruit and vegetable intake, high intake of cereals, and excessive meat and egg intake were associated with a higher incidence of GERD. In addition, some studies showed that vegetables, fruits, and high-fiber diets are inversely related to GERD [26]; however, a Sweden monozygotic twin-based study revealed that none of the aforementioned items was linked to the GERD symptoms [25].

However, to the best of our knowledge, the effect of the DASH diet on GERD and its symptoms has not been investigated. The purpose of this study was to evaluate the association between a DASH-style diet and GERD symptoms in adolescents.

## Materials and methods

This study was done in the context of a larger cross-sectional (the GAN 2020) [27–29], conducted on Iranian adolescents aged 13–14 years from February 2020 to June 2020 in Yazd, Iran. Study participants were recruited from 48 schools. The schools were chosen among all public and private schools in urban areas of Yazd city through cluster sampling and all students with the inclusion criteria were recruited.

The GAN questionnaire was translated into Persian, and its face validity was evaluated by experts. The reliability of the translated version was confirmed by a study conducted on 100 selected students aged 13 to 14 years using Cronbach’s alpha. The alpha coefficient for rhinitis and eczema symptoms turned out to be 0.74, which shows an appropriate internal consistency.

Electronic socio-demographic, dietary intake, and GERDQ questionnaires were provided to students in collaboration with school principals. Among all 13–14 students from selected schools, volunteer students completed the questionnaires. Before completion of the questionnaires, informed consent through the electronic form was approved by all participants. Out of 7214 students, 5141 completed the questionnaire (71.3% response rate). Ambiguous demographic information was re-checked through phone contact.

### Dietary intake assessment

Dietary intake data were collected by a food questionnaire which included 26 list of food items with standard serving sizes. The participants were asked about the consumption frequency of each item in the food questionnaire during the last year, and the frequency of intake (never or very low/ one or two times per week/ often or every day) according to the food type. In a previous study, the validity and reliability of this questionnaire were confirmed [29].

### Calculating DASH-style diet adherence score

The DASH dietary pattern scores were computed by adopting the method proposed by Fung et al. [30]. Food items/groups used for calculating the adherence score were fruits, vegetables, nuts and legumes, dairy, grains, red and processed meat, and sweetened beverages. In such a way, individuals who consumed never or very low, one or two times per week, often or every day of fruits, vegetables, nuts and legumes, dairy were received 1, 2, and 3 points, respectively. The intake of grains, red and processed meats, and sweetened beverages received were reversely scored. The scores were then summed up to compute the total score. Therefore, the score for each participant ranged from 7 to 21 (**Supplementary Table **1). A greater score showed greater adherence to the DASH-style diet. Then, participants were categorized into tertiles based on the scores.

### Diagnosis of gastroesophageal reflux disease and its symptoms

The participants were asked to recall their GERD symptoms and the frequency of their occurrence over the last week using the validated GERD questionnaire [31]. The questionnaire had six components: four positive predictors of GERD with a 4-grade Likert scale including frequency of heartburn, regurgitation, sleep disturbance and the use of over-the-counter medications for these symptoms, and also two negative predictors scored with a reversed Likert scale including stomach pain and nausea. A full explanation in simple and understandable terms was provided at the beginning of the reflux questionnaire. The GERDQ score is determined through the sum of the scores and ranged from 0 to 8 for each item [31]. The sum of the points for the aforementioned frequencies was considered as a participant’s GERDQ score, and a diagnosis of GERD could be possible if the sum of all scores was ≥ 8 points [31].

### Assessment of other variables

Weight was measured to the nearest 100 g using digital scales with minimal clothes while no wearing shoes. Height was measured to the nearest 0.5 cm by a tape meter fixed on a wall while the subjects were without shoes and standing in a normal position. Watching television and computer use (2–4 h/ 5–8 h/ 9–14 h a day) also, were obtained using a self-reported online GAN questionnaire.

### Statistical analysis

Statistical analysis was performed using the statistical package for social sciences (SPSS, version 23, IBM). The normality of data was tested by the Kolmogorov-Smirnov method. Study participants were categorized to tertiles based on DASH-style diet scores. The comparison of quantitative and qualitative values across the tertiles of DASH diet scores was performed using one-way analysis of variance with Bonferroni post hoc test and chi-square test, respectively. Dietary food groups and the intakes of nutrients were compared across DASH-style diet tertiles by using the analysis of covariance (ANCOVA) with Bonferroni correction. The odds of GERD and its symptoms were compared between those with the lowest DASH-style diet score and those with higher adherence to the diet using logistic regression in crude model and multivariable-adjusted models. Participants’ sex (male as reference group), age (continuous variable), watching TV and computer use (use of TV & computer for 2–4 h as reference group), smoking (“none” used as reference group), and body mass index (BMI) (continuous variable) were considered as confounding variables. Participants’ sex and age were adjusted in model I. Watching TV and computer use and smoking were further adjusted in model II, and finally, in model III, the association was additionally controlled for BMI. A p-value of < 0.05 was considered statistically significant.

## Results

From 5,141 participants aged 13–14 who were included in the current study, 153 individuals had GERD. For GERD symptoms, 107, 99 and 198 participants had reflux, heartburn, and stomach pain, respectively. The characteristics of the study population across the tertile of the DASH-style diet score are shown in Table 1. The DASH-style diet score ranged from 8 to 19 in this population. There were significant differences in ethnicity (P = 0.005), physical activity (P < 0.001), and smoking (P < 0.001) of participants across tertiles of the DASH-style diet score. Subjects with the highest DASH style diet score had lower reflux (P < 0.001), heartburn (P = 0.009), nausea (P = 0.009), and stomach pain (P < 0.001).

**Table 1:** General characteristics of the subjects according to the tertile of DASH style diet adherence

Variables		Tertile of DASH style diet score					P-value
		T1 (n = 2023, 8–14)		T2 (n = 1431, 15)		T3 (n = 1687, 16–19)	
Sex							
Male		835 (41.2)		573 (44.5)		664 (39.3)	0.48
Female		1188 (58.7)		858 (59.9)		1023 (60.6)	
Age (years)		13.3 ± 0.46		13.3 ± 0.47		13.3 ± 0.46	0.17
BMI (kg/m^2)^		20.6 ± 4.05		20.6 ± 4.1		20.6 ± 4.0	0.98
Boys (13 years old)							
Obesity (BMI > 24.8)		68 (8.14)		43 (7.50)		53 (7.98)	
Overweight (BMI > 20.8)		163 (19.52)		100 (17.45)		148 (22.29)	
Normal (16.4–20.8)		218 (26.11)		134 (23.39)		165 (24.85)	
Thinness (BMI < 16.4)		49 (5.87)		46 (8.03)		36 (5.42)	
Sever thinness (BMI < 14.9)		37 (4.43)		28 (4.89)		37 (5.57)	0.37
Boys (14 years old)							
Obesity (BMI > 25.9)		22 (2.63)		16 (2.79)		24 (3.61)	
Overweight (BMI > 21.8)		85 (10.18)		67 (11.69)		80 (12.05)	
Normal (17-21.8)		147 (17.60)		106 (18.50)		91 (13.70)	
Thinness (BMI < 17)		26 (3.11)		21 (3.66)		16 (2.41)	
Sever thinness (BMI < 15.5)		20 (2.40)		12 (2.09)		14 (2.11)	0.45
Girls (13 years old)							
Obesity (BMI > 26.2)		58 (4.88)		41 (4.78)		52 (5.08)	
Overweight (BMI > 21.8)		280 (23.57)		199 (23.19)		213 (20.82)	
Normal (16.6–21.8)		361 (30.39)		255 (29.72)		328 (32.06)	
Thinness (BMI < 16.6)		71 (5.98)		61 (7.11)		74 (7.23)	
Sever thinness (BMI < 14.9)		66 (5.56)		34 (3.96)		56 (5.47)	0.5
Girls (14 years old)							
Obesity (BMI > 27.3)		18 (1.52)		23 (2.68)		13 (1.27)	
Overweight (BMI > 22.7)		110 (9.26)		69 (8.04)		80 (7.82)	
Normal (17.2–22.7)		154 (12.96)		127 (14.80)		150 (14.66)	
Thinness (BMI < 17.2)		47 (3.96)		36 (4.20)		39 (3.81)	
Sever thinness (BMI < 15.4)		23 (1.94)		13 (1.52)		18 (1.76)	0.33
Ethnicity							0.005
Kord		9 (0.44)		3 (0.21)		15 (0.89)	
Turk		22 (1.09)		6 (0.42)		12 (0.71)	
Persian		1958 (96.7)		1394 (97.4)		1629 (96.5)	
Lor		9 (0.44)		13 (0.91)		15 (0.89)	
Arab		12 (0.59)		12 (0.84)		14 (0.83)	
Balooch		13 (0.64)		3 (0.21)		2 (0.12)	
Physical activity (watching TV and computer use)							< 0.001
2–4 h		827 (40.8)		722 (50.4)		947 (56.1)	
5–8 h		748 (36.9)		482 (33.6)		546 (32.3)	
9–14 h		448 (22.1)		227 (15.8)		194 (11.5)	
Smoking							
Yes		63 (3.12)		17 (1.19)		14 (0.83)	
No		1960 (96.8)		1414 (98.81)		1673 (99.17)	< 0.001
reflux							
Yes		63 (3.1)		24 (1.6)		20 (1.1)	< 0.001
No		1960 (96.8)		1407 (98.3)		1667 (98.8)	
heartburn							
Yes		52 (2.5)		16 (1.1)		31 (1.8)	0.009
No		1971 (97.4)		1415 (98.8)		1656 (98.1)	
nausea							
Yes		39 (1.9)		13 (0.91)		16 (0.95)	0.009
No		1984 (98.0)		1418 (99.0)		1671 (99.0)	
stomach pain							
Yes		101 (4.9)		44 (3.0)		53 (3.1)	< 0.001
No		1922 (95.0)		1387 (96.9)		1634 (96.8)	

Values are mean ± SD or number (percentages)

^a^χ^2^ Test for ordinal qualitative variables and t-test for continuous variables

The daily intake of dietary food groups of participants across DASH-style score tertile is shown in Table 2. It was revealed that the consumption frequency of fruits (P < 0.001), vegetables (P < 0.001), legumes (P < 0.001), nuts (P < 0.001), dairy (P < 0.001), grains (P < 0.001), meats (P < 0.001), processed meats (P < 0.001), sweets (P < 0.001), and sweetened beverages (P < 0.001) are significantly different across tertiles of the DASH-style diet score.

**Table 2:** The daily intake of dietary food groups of subjects according to the tertile of DASH style diet adherence

Variables		Tertile of DASH score						
		T1 (n = 3148) (Median score = 14)		T2 (n = 2101) (Median score = 15)		T3 (n = 2418) (Median score = 16)		P-value
**Fruits**								
Never (%)		133 (6.5)		10 (0.70)		2 (0.12)		< 0.001
Weekly		636 (31.4)		182 (12.7)		72 (4.2)		
Every day		1254 (61.9)		1239 (86.5)		1613 (95.6)		
**Vegetables**								
Never		1020 (50.4)		212 (14.8)		51 (3.0)		< 0.001
Weekly		949 (46.9)		1000 (69.8)		813 (48.1)		
Every day		429 (21.2)		219 (15.3)		823 (48.7)		
**Legumes**								
Never		103 (5.0)		12 (0.84)		9 (0.53)		< 0.001
Weekly		1491 (73.7)		844 (58.9)		514 (30.4)		
Every day		574 (18.2)		577 (40.1)		1164 (69.0)		
**Nuts**								
Never		1114 (55.0)		670 (46.8)		690 (40.9)		< 0.001
Weekly		822 (40.6)		657 (45.9)		813 (48.1)		
Every day		87 (4.3)		104 (7.2)		184 (10.9)		
**Dairy**								
Never		82 (4.0)		5 (0.35)		5 (0.30)		< 0.001
Weekly		868 (42.9)		267 (18.6)		136 (8.0)		
Every day		1073 (53.0)		1159 (80.9)		1546 (91.6)		
**Grains**								
Never		13 (0.64)		1 (0.07)		3 (0.18)		< 0.001
Weekly		199 (9.8)		149 (10.4)		258 (15.2)		
Every day		1811 (89.5)		1281 (89.5)		1426 (84.5)		
**Meat**								
Never		134 (6.6)		67 (4.6)		103 (6.1)		0.14
Weekly		947 (46.8)		660 (46.1)		778 (46.1)		
Every day		942 (46.5)		704 (49.2)		806 (47.7)		
**Processed meats**								
Never		898 (44.3)		777 (54.3)		1113 (65.9)		< 0.001
Weekly		985 (48.6)		616 (43.0)		562 (33.3)		
Every day		140 (6.9)		38 (2.6)		12 (0.71)		
**Sweets**								
Never		299 (14.7)		270 (18.8)		574 (34.0)		< 0.001
Weekly		1059 (52.3)		868 (60.6)		1008 (59.7)		
Every day		665 (32.8)		293 (20.4)		105 (6.2)		
**Sweetened Beverages**								
Never		813 (40.1)		712 (49.7)		1133 (67.1)		< 0.001
Weekly		829 (40.9)		608 (42.4)		517 (30.6)		
Every day		381 (18.3)		111 (7.7)		37 (2.1)		

Values are number and percentages

The associations between adherence to a DASH-style diet and reflux, heartburn, nausea, and pain in the stomach are shown in Table 3. A significant inverse association was seen between the greatest adherence to the DASH-style diet and reflux, nausea, and pain in the stomach in the crude model. This association remains significant even after controlling for potential confounders as subjects in the highest DASH-style diet tertile were 57%, and 29% less likely to have reflux, and pain in the stomach, respectively. For nausea after controlling for potential confounders, a trend toward a significant inverse association was seen between the DASH-style diet and the odds of nausea. However, no significant relationship was observed between adherence to a DASH-style diet and heartburn.

**Table 3:** Association between a DASH style diet and reflux, heartburn and stomach pain

DASH style diet score								
		T1		T2		T3		P _trend_
		OR (95% CI)		OR (95% CI)		OR (95% CI)		
No. with/without reflux		63/1960		24/1407		20/1667		
Crude		1.00		0.53 (0.32–0.85)		0.37 (0.22–0.61)		**< 0.001**
Model 1		1.00		0.53 (0.33–0.85)		0.37 (0.22–0.61)		**< 0.001**
Model 2		1.00		0.59 (0.36–0.95)		0.43 (0.26–0.73)		**0.001**
Model 3		1.00		0.59 (0.36–0.95)		0.43 (0.26–0.73)		**0.001**
No. With/without heartburn		52/1971		16/1415		31/1656		
Crude		1.00		0.42 (0.24–0.75)		0.70 (0.45–1.11)		0.08
Model 1		1.00		0.42 (0.24–0.75)		0.70 (0.45–1.11)		0.08
Model 2		1.00		0.50 (0.28–0.89)		0.88 (0.55–1.40)		0.44
Model 3		1.00		0.50 (0.28–0.88)		0.88 (0.55–1.40)		0.44
No.with/without nausea		39/1984		13/1418		16/1671		
Crude		1.00		0.46 (0.24–0.87)		0.48 (0.27–0.87)		**0.008**
Model 1		1.00		0.45 (0.24–0.86)		0.48 (0.26–0.86)		**0.007**
Model 2		1.00		0.53 (0.28–1.01)		0.59 (0.32–1.08)		**0.05**
Model 3		1.00		0.53 (0.28–1.01)		0.59 (0.32–1.08)		**0.05**
No. with/without stomach pain		101/1922		44/1387		53/1634		
Crude		1.00		0.60 (0.42–0.86)		0.61 (0.43–0.86)		**0.003**
Model 1		1.00		0.59 (0.41–0.85)		0.60 (0.43–0.85)		**0.002**
Model 2		1.00		0.66 (0.45–0.95)		0.71 (0.50-1.00)		**0.03**
Model 3		1.00		0.66 (0.45–0.95)		0.71 (0.50-1.00)		**0.03**

Model 1: Adjusted for age and sex (for total participants)

Model 2: Further adjusted for watch TV & computer use and smoking

Model 3: Additionally, adjustment for BMI

The relationship between GERD and adherence to the DASH-style diet was assessed in the whole population and each sex (Table 4). Subjects in higher tertile of DASH-style diet were 55% less likely to have GERD [odds ratio (OR) = 0.45; 95% confidence interval (CI): 0.30–0.68, P trend < 0.001). After adjustment for potential confounders, this association remained significant (OR = 0.51; 95%CI: 0.34–0.77, P trend < 0.01). Moreover, after the subgroup of analysis based on sex, boys in the highest tertile of DASH-style diet score had 62% (OR = 0.38; 95%CI: 0.19–0.77, P trend = 0.002) lower odds of GERD in the fully adjusted model. In addition, a trend toward a significant inverse association was seen between a DASH-style diet and the odds of GERD among girls.

**Table 4:** Association between DASH style diet adherence and GERD

DASH style diet score								
		T1		T2		T3		P _trend_
		OR (95% CI)		OR (95% CI)		OR (95% CI)		
Girls								
No. with/without GERD		50/1138		24/834		23/1000		
Crude		1.00		0.65 (0.39–1.07)		0.52 (0.31–0.86)		**0.009**
Model 1		1.00		0.65 (0.39–1.07)		0.52 (0.31–0.86)		**0.009**
Model 2		1.00		0.73 (0.44–1.21)		0.61 (0.36–1.01)		**0.05**
Model 3		1.00		0.73 (0.44–1.21)		0.61 (0.37–1.01)		**0.05**
Boys								
No. with/without GERD		37/897		8/565		11/653		
Crude		1.00		0.30 (0.14–0.66)		0.36 (0.18–0.71)		**0.001**
Model 1		1.00		0.30 (0.14–0.66)		0.36 (0.18–0.71)		**0.001**
Model 2		1.00		0.32 (0.14–0.69)		0.39 (0.19–0.79)		**0.003**
Model 3		1.00		0.32 (0.14–0.70)		0.38 (0.19–0.77)		**0.002**
Total population								
No. with/without GERD		87/1936		32/1399		34/1653		
Crude		1.00		0.50 (0.33–0.76)		0.45 (0.30–0.68)		**< 0.001**
Model 1		1.00		0.50 (0.33–0.76)		0.45 (0.30–0.68)		**< 0.001**
Model 2		1.00		0.55 (0.36–0.84)		0.51 (0.34–0.77)		**0.001**
Model 3		1.00		0.55 (0.36–0.83)		0.51 (0.34–0.77)		**0.001**

Model 1: Adjusted for age and sex (for total participants)

Model 2: Further adjusted for watch TV & computer use and smoking.

Model 3: Additionally, adjustment for BMI

## Discussion

To the best of our knowledge, this is the first study on association between DAS-style diet adherence and GERD and its typical symptoms. We found that the highest adherence to the DASH style diet is inversely associated with risk of GERD in adolescents, and also some GERD symptoms including, reflux, and pain in the stomach. However, no significant association was observed between the DASH-style diet and heartburn.

The DASH diet is characterized by high levels of food groups that are regarded as protective components against GERD such as vegetables, fruits, legumes, whole grains, and white meats, and also low levels of food items related to an increased GERD risk including red/processed meats, salt, sweets, and beverages [10, 14, 32, 33]. Consistence with our findings, HassanzadehKeshteli et al. [34] found inverse associations between the intake of fruits and vegetables and the risk of GERD among adults. Wu et al. [12] also provided evidence supporting the association between a high intake of fruits and vitamin C and the prevention of reflux. The DASH diet is rich in antioxidants such as vitamin C, vitamin E, and ß-carotene contained in fruits, vegetables, nuts, and whole grains [35–37]. It has been demonstrated that oxidative stress is contributed to the development of GERD [38, 39]; moreover, diets rich in vitamin C were related to a lower GERD risk [40]as some investigations demonstrated that high vitamin C intake has a protective effect on GERD [13, 40]. On the other hand, previous evidence recommended the consumption of alkaline-forming food items such as white meat, raisins, bananas, grapes, lemons, maple fruit, molasses, and all types of vegetables [41]. These foods neutralize body acids thus reducing alterations for the development of heartburn. Sulphur and Phosphorus act as buffers to maintain pH [42].

High content of dietary fiber in the DASH-style diet might also play an important role in reducing GERD risk [43, 44]. It has been shown that dietary fibers scavenge gastric nitrites and reduce the substrate availability for non-enzymatic nitric oxide synthesis, thereby, reducing the concentration of nitric oxide in the gastroesophageal junction and therefore, preventing reflux [45]. Decreasing the gastric nitrites can also relax the low esophageal sphincter (LES) [46]. In addition, buffering capacity of some dietary fibers can decrease the acidity of gastric content [47].

DASH diet recommends people to consume low levels of salt and previous evidence has shown that an increase in salt intake is associated with GERD [14, 48]. Higher salt intake is attributed to delayed gastric emptying and increased pancreatic biliary secretion after a high intake of salt [49]. On the other hand, sweets can also cause reflux because of their high osmolality [50]. In some studies, chocolate has been shown to develop reflux by reducing the pressure of LES [51, 52].

A large number of participants is one of the strengths of the current study. However, our study had several limitations. The cross-sectional nature of our study is unable to approve the causal relationship between a DASH-style diet and GERD and this limitation could affect the findings of the present study. In our study, GERD was diagnosed by a symptom-based questionnaire, while some patients with GERD may present with atypical symptoms or even some patients experience no symptoms [53, 54]. Additionally, the estimation of individual’s dietary intakes by using the FFQ is prone to misreporting and misclassification of participants. Since the current study was only conducted on adolescents aged 13–14 years, the generalizability of findings to other age groups would be limited; therefore, it should be considered that the present findings might be different among other age categories.

## Conclusion

In conclusion, our study revealed that adherence to the DASH diet may has protective role in GERD and GERD symptoms including, reflux, nausea, and stomach pain in adolescents. The role of DASH diet in GERD risk and clinical symptoms in adolescents warrants further investigation. Large prospective studies and controlled clinical trials are needed to confirm the effectiveness of the DASH diet in the prevention and management of GERD in adolescents, respectively.

## Electronic supplementary material

Below is the link to the electronic supplementary material.

Supplementary Material 1

## Data Availability

The dataset of the present study is available from the corresponding author on reasonable request.

## List of Abbreviations

GERD: Gastroesophageal reflux disease
DASH: Dietary approaches to stop hypertension
GI: gastrointestinal
BMI: body mass index
OR: odds ratio
CI: confidence interval
LES: low esophageal sphincter
