# Supplementary material for: Dietary approaches to stop hypertension (DASH)-style diet in association with gastroesophageal reflux disease in adolescents
Source: BMC Public Health. 2023 Feb 17;23:358. doi: 10.1186/s12889-023-15225-6 (PMC9936743; doi:10.1186/s12889-023-15225-6)
Supplement: Supplementary file 1 — Supplementary Material 1 [file 12889_2023_15225_MOESM1_ESM.docx]

| **Supplementary Table 1- Method used for calculating DASH adherence score** | | | |
| --- | --- | --- | --- |
| Food groups | Never or very low | Once or twice per week | Often or every day |
| Fruits | 1 | 2 | 3 |
| Vegetables | 1 | 2 | 3 |
| Nuts and legumes | 1 | 2 | 3 |
| Dairy | 1 | 2 | 3 |
| Grains | 3 | 2 | 1 |
| Red and processed meat | 3 | 2 | 1 |
| Sweetened and beverages | 3 | 2 | 1 |
